# Some of the Newest Therapeutic Methods in Canine Atopic Dermatitis

**DOI:** 10.3390/vetsci13040403

**Published:** 2026-04-20

**Authors:** Constantin-Cătălin Mazilu, Anamaria-Hortensia Strichea, Gheorghe Solcan

**Affiliations:** Internal Medicine Clinic, Faculty of Veterinary Medicine, Iași University of Life Sciences, 8 M. Sadoveanu Alley, 700489 Iași, Romania; mazilu.cata@yahoo.com (C.-C.M.); anamaria.strichea@iuls.ro (A.-H.S.)

**Keywords:** canine atopic dermatitis, atopy, cAD, cAD therapy

## Abstract

Canine atopic dermatitis is a hereditary chronic inflammatory and pruritic skin disease, which is mediated by T cells and requires long-term, individualized management. In recent years, numerous studies have described a wide range of therapeutic approaches for canine atopic dermatitis, including fast-acting symptomatic treatments, long-term immune-modulating interventions, and strategies to support skin barrier function and microbial balance. This review summarizes the principal treatment modalities currently available, including glucocorticoids, cyclosporine A, mycophenolate, Janus kinase inhibitors, lokivetmab, and allergen-specific immunotherapy, as well as complementary strategies aimed at restoring skin barrier integrity. Emphasis is placed on the importance of a multimodal and personalized approach to optimize long-term disease control and improve quality of life in affected dogs. Providing an integrated overview of current evidence, this article aims to guide clinicians in making informed, evidence-based decisions and to support the safe and effective management of canine atopic dermatitis.

## Simple Summary

Canine atopic dermatitis is a chronic inflammatory and pruritic skin disease with genetic predisposition that requires long-term, individualized management. Symptomatic therapies provide rapid relief but may be limited by long-term adverse effects, particularly with corticosteroids. Disease-modifying interventions, such as targeted antibodies and allergen-specific immunotherapy, offer sustained control of clinical signs and the potential for long-term remission. Adjunctive strategies that enhance skin barrier integrity and microbial homeostasis can further improve outcomes and reduce reliance on systemic medications.

## 1. Introduction

**Figure 1:**
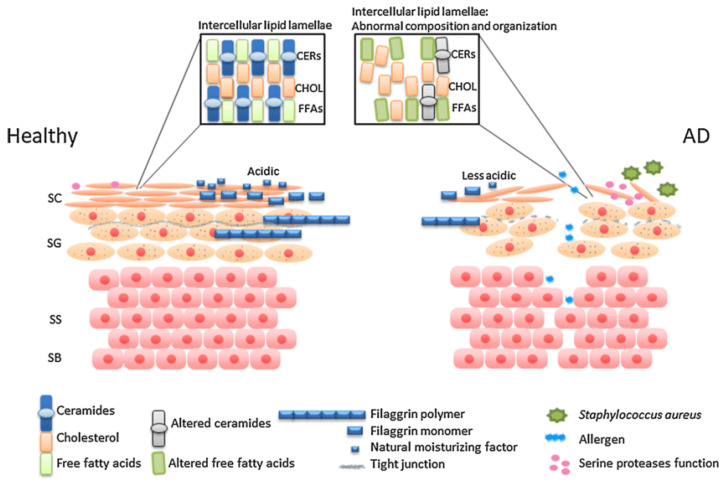
Skin barrier in healthy and atopic people [17].

According to the International Committee on Allergic Diseases of Animals [1], canine atopic dermatitis (cAD) is a hereditary, primarily pruritic, T-cell-mediated inflammatory skin disorder in which disease development results from the interaction between skin barrier defects, allergen sensitization, and microbial dysbiosis [1]. It has been hypothesized that the skin barrier is disrupted in dogs with atopic dermatitis, with notable similarities to human disease [2] in terms of barrier impairment (Figure 1). Current evidence indicates that this dysfunction is multifactorial, involving both structural and biochemical alterations of the stratum corneum. These include disruptions in lipid organization, quantitative and qualitative changes in ceramides, increased transepidermal water loss, and abnormalities in structural proteins such as filaggrin. Notably, several of these defects are also present in non-lesional skin, suggesting a more generalized impairment rather than a purely inflammation-driven process [3].

Even in non-lesional skin, research has documented disorganization of intercellular lipid lamellae [4] and a marked reduction in total ceramides [5,6], including specific subclasses [7,8], which contributes to increased transepidermal water loss. Experimental work further shows that allergen exposure exacerbates these lipid abnormalities [8], while other investigations [9] report decreased levels of the anti-inflammatory metabolite sphingosine-1-phosphate, potentially reflecting enhanced enzymatic degradation. Collectively, these findings suggest that lipid and ceramide disturbances compromise barrier integrity and may facilitate both allergen penetration and inflammatory amplification, although it remains unclear whether they represent a primary defect or arise secondarily. Strategies aimed at restoring barrier integrity, such as topical ceramide-based formulations or oral supplementation with essential fatty acids, are currently under evaluation in affected dogs. Similar interventions have already been tested in human atopic dermatitis [10,11,12], supporting the translational potential of barrier-targeted therapies. While further research is needed to determine optimal protocols and efficacy, these approaches offer a promising avenue for improving disease management by directly addressing underlying barrier defects.

Filaggrin has been widely studied in human AD, where gene mutations represent key but not universal risk factors. In dogs, epidermal filaggrin is reduced or absent in some AD cases, though gene mutations are generally not implicated. These findings suggest breed- and region-specific roles, but the precise contribution of filaggrin to AD pathogenesis remains unclear [2,7,8,13]. This disruption may contribute to disease pathogenesis by increasing susceptibility to allergic sensitization. Advances in bioinformatics and high-throughput sequencing have transformed the characterization of the skin microbiome, enabling comprehensive identification of bacterial communities and analysis of shifts in diversity and population structure. While healthy human skin exhibits a highly diverse and site-specific microbiome, atopic dermatitis is associated with microbial imbalances that may contribute to disease development [14]. Allergen exposure in sensitized dogs has been shown to alter the skin microbiome, with a notable increase in *Staphylococcus pseudintermedius* at lesion sites. This suggests that immune activation may drive microbial imbalance, potentially worsening disease manifestations [15,16]. However, despite multiple studies documenting significant dysbiosis in the skin and external ear canals of dogs with atopic dermatitis, a direct causal link between microbial imbalance and disease development has not yet been established. Staphylococcal predominance is consistently reported, yet it remains unclear whether these changes drive pathology or arise secondary to barrier defects and inflammation. Notably, one of the most recent reviews [2] emphasizes that although the association is well documented, the precise role of microbial dysbiosis in disease pathogenesis remains unresolved.

The aim of this review is to synthesize the evidence published until 2026 regarding the efficacy and safety of current therapeutic options for cAD, and to identify knowledge gaps that justify further research.

## 2. Article Selection

A systematic search of Google Scholar, PubMed, and Scopus was conducted to identify studies on canine atopic dermatitis published between January 2000 and January 2026. Initially, a broad search was performed using the keyword “canine atopic dermatitis,” followed by targeted searches focusing on specific therapeutic interventions, including glucocorticoids, cyclosporine, mycophenolate mofetil, JAK inhibitors, lokivetmab, allergen-specific immunotherapy, and topical formulations supporting the skin barrier.

Inclusion criteria were as follows: peer-reviewed studies, clinical trials, observational studies, and reviews that investigated treatment efficacy, safety, or skin barrier restoration in dogs with atopic dermatitis. Studies conducted on small animal cohorts were also included for their potential to guide future research, and this was explicitly noted in the text. Exclusion criteria included non-English articles, studies on other species, conference abstracts without full texts, and studies not focused on clinical outcomes.

**Figure 2:**
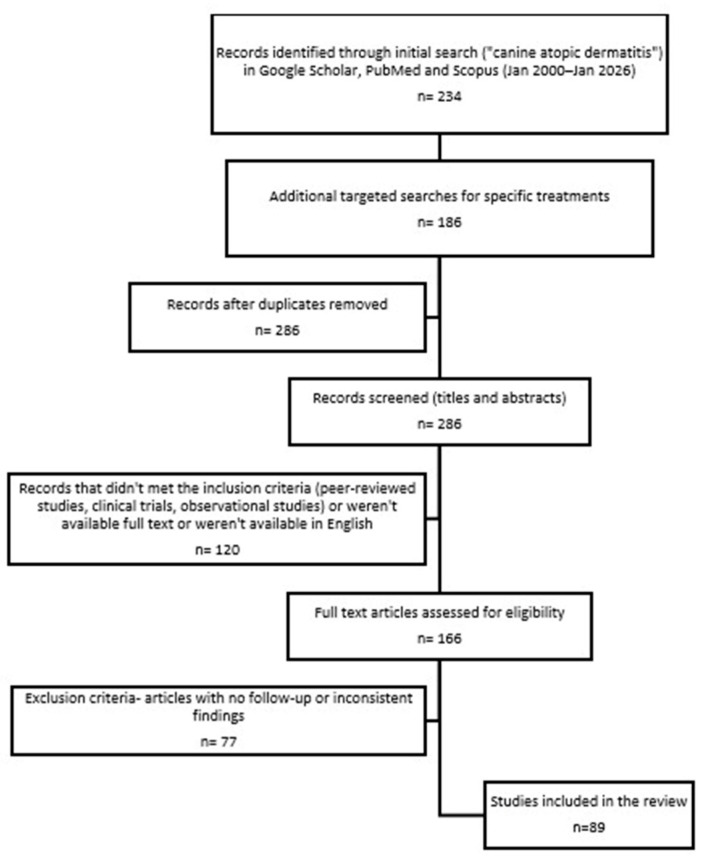
PRISMA style flow diagram with article selection.

Titles and abstracts were screened for relevance, and the full texts of selected articles were reviewed to extract data on treatment outcomes and clinical applicability. The selection process followed PRISMA guidelines [18] to ensure methodological rigor and transparency (Figure 2).

When two or more studies addressed the same aspect of canine atopic dermatitis therapy, we selected the study with higher statistical power (larger sample size or more precise estimates) and stronger scientific quality (rigorous study design and lower risk of bias). Studies with lower statistical power or methodological limitations were considered only when no higher-quality evidence was available.

## 3. Overview of Therapeutic Strategies

The management of cAD patients involves:

- identifying and addressing (or, if possible, avoiding) the associated flare factors (flea salivary antigens, antibacterial/antifungal interventions, identifying and avoiding food allergens and environmental allergens from dust mites and pollens);
- using of topical and/or systemic treatment to decrease inflammation and pruritus [19]; glucocorticoids, cyclosporin A, mycophenolate mofetil, Janus Kinase inhibitors, biologic therapies directed against key cytokines (Lokivetmab);
- long-term management of the disease (allergen-specific immunotherapy);
- complementary therapeutic approaches.

### 3.1. Glucocorticoids

#### 3.1.1. Systemic Glucocorticoids

Widely used for their rapid therapeutic efficacy, glucocorticoids exert strong anti-inflammatory and immunosuppressive effects by regulating gene transcription. Through direct action on target genes, these agents suppress the synthesis of pro-inflammatory mediators while promoting the expression of anti-inflammatory cytokines, ultimately modulating both humoral and cell-mediated immune responses [20,21].

In clinical practice, oral glucocorticoids remain a cornerstone in the treatment of cAD, with prednisolone, prednisone, and methylprednisolone typically administered at daily doses of 0.5–1 mg/kg [20]. Evidence indicates that glucocorticoid therapy leads to a rapid alleviation of pruritus in affected animals. Following oral administration, clinical improvement was observed as early as four hours in approximately 25% of cases, while a therapeutic response within 3–7 days was reported in up to 90% of animals. These outcomes were achieved using initial prednisolone regimens of 0.5–1 mg/kg administered once or twice daily for 3–7 days, as well as methylprednisolone at doses of 0.4–0.8 mg/kg once daily for 5–7 days. Additionally, a single intramuscular administration of dexamethasone (0.2 mg/kg) resulted in a noticeable antipruritic effect within one hour [22]. In contrast, several investigations initiated clinical evaluation only after two weeks or more, precluding accurate assessment of treatment onset. Across studies employing comparable starting doses of prednisolone, methylprednisolone, or single-dose intramuscular dexamethasone, the magnitude of pruritus reduction and improvement in clinical scores varied considerably, reflecting differences in study design and outcome measures. Overall, these dosing protocols were associated with an approximate 50% reduction in lesion severity in 50% to 80% of dogs and a comparable decrease in pruritus intensity in 42% to 70% of treated animals, with higher response rates generally reported in studies with stronger levels of evidence [22,23,24,25,26].

**Figure 3:**
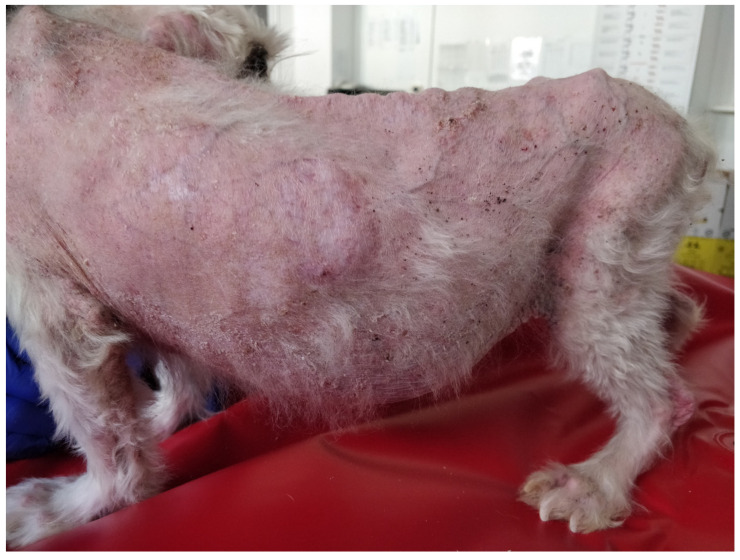
Iatrogenic Cushing’s syndrome. Pendulous abdomen, extensive alopecia, punctate cutaneous calcinosis, and increased skin fragility.

**Figure 4:**
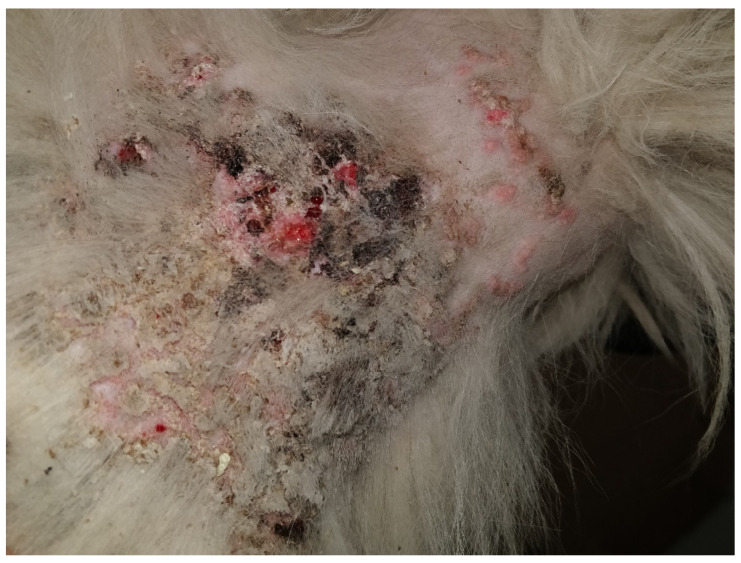
Iatrogenic Cushing’s syndrome. Calcinosis cutis in a dog following prolonged corticosteroid administration.

Despite their effectiveness, glucocorticoid therapy requires careful management, as adverse effects may arise, particularly with prolonged administration. The likelihood and severity of these effects are closely associated with steroid potency, dosage, and treatment duration. To improve long-term disease control while minimizing complications, extended dosing intervals such as alternate-day regimens or administration every 48 to 72 h are recommended. Documented adverse reactions include clinical signs of hypercortisolism, including polydipsia, polyuria, polyphagia, weight gain, dermal atrophy (Figure 3), calcinosis cutis (Figure 4), secondary pyoderma, panting, urinary tract infections, abdominal distension, depression, and diarrhea [22,27,28,29]. These limitations highlight the urgent need for safe and effective alternative therapies for the lifelong management of canine atopic dermatitis.

#### 3.1.2. Topical Glucocorticoids

In canine patients, topical corticosteroids play a central role in achieving remission during acute disease exacerbations and in the management of localized lesions. DeBoer advocated the use of spray-formulated topical corticosteroids, such as triamcinolone acetonide 0.015%, which provide effective anti-inflammatory activity at relatively low drug concentrations while maintaining a favorable safety profile and a reduced likelihood of adverse effects [30]. Potent topical glucocorticoids may induce adverse cutaneous effects, such as the formation of pigmented viral plaques, which can regress after discontinuation of therapy but recur upon re-exposure, highlighting the need for careful monitoring during treatment [31].

In cases of otitis, proactive use of mometasone appears not to increase the risk of secondary microbial overgrowth, with observed clinical benefits supported by cytological and otoscopic improvements [32]. Another safe and effective option for both reactive and proactive management of non-infectious chronic otitis externa is the use of hydrocortisone aceponate, which demonstrated efficiency in reducing recurrence rates [33,34]. A 7–14-day course of a commercial hydrocortisone aceponate diester glucocorticoid ear spray was safe and yielded improvements comparable to an antibiotic–antifungal–glucocorticoid formulation, controlling both inflammation and microbial overgrowth. These findings suggest that even when cytology detects microbial overgrowth, a topical glucocorticoid spray alone may serve as an effective first-line therapy—a valuable consideration amid rising antimicrobial resistance [35].

Topical corticosteroid therapy is effective primarily for localized lesions, such as those seen in otitis, whereas multifocal or generalized atopic dermatitis typically requires identification of an appropriate systemic treatment. Daily application of steroids to localized skin lesions for two to four weeks, eventually used intermittently (two to three times weekly) on the previously affected skin, even if lesions are no longer visible as proactive treatment strategy is associated with a lower incidence of flare-ups and a longer duration of remission when contrasted with reactive therapy, which involves treatment only when clinical signs are apparent [19].

### 3.2. Cyclosporin A (csA)

#### 3.2.1. Systemic Use

Cyclosporine is a lipophilic cyclic polypeptide derived from the fungal species *Tolypocladium inflatum* (Gams), initially developed for the prevention of organ transplant rejection. Despite its potent immunosuppressive properties, cyclosporine exhibits relatively low cytotoxicity. Its primary mechanism of action involves inhibition of IL-2 gene transcription and suppression of T cell responsiveness to IL-2, resulting in impaired activation of both T-helper and cytotoxic T lymphocytes. In addition, cyclosporine downregulates interferon-α (IFN-α) transcription, thereby attenuating macrophage and monocyte activation signals. The synthesis of several other cytokines, including IL-3, IL-4, IL-5, tumor necrosis factor-α (TNF-α), and interferons, may also be reduced. Through these combined effects, cyclosporine interferes with multiple immune pathways, leading to inhibition of mononuclear cell function, antigen presentation, mast cell and eosinophil activity, histamine and prostaglandin release, neutrophil adhesion, natural killer cell activity, and B cell growth and differentiation. In the context of atopic dermatitis, an additional proposed mechanism involves suppression of mast cell degranulation by modulating mast cell–nerve interactions. Furthermore, cyclosporine has been shown to directly inhibit histamine release from canine mast cells [36].

To reach optimal clinical effectiveness, cyclosporine A should be administered at 5 mg/kg per day for about four weeks (induction period), after which the dosage may be gradually decreased [37]. The results of a large-scale study demonstrated that CsA monotherapy was as effective as methylprednisolone for the treatment of cAD. The study further showed that the initial induction dose of 5 mg/kg could be gradually reduced over time without compromising clinical efficacy, with maintenance doses being at least half of those used during induction [26]. In dogs affected by atopic dermatitis, a brief initial course of prednisolone accelerated the therapeutic effects of CsA in alleviating pruritus and associated clinical manifestations. Reported adverse events were consistent with those typically associated with each of the administered veterinary medicinal products [37].

Clinical studies had previously demonstrated the efficacy of CsA in the management of cAD. These trials were subsequently evaluated in a systematic review of pharmacological interventions for AD in dogs. The review concluded that CsA administered at 5 mg/kg once daily for up to 16 weeks produced consistently high therapeutic responses. Notable reductions in pruritus and skin lesions were observed after four weeks of treatment. In approximately half of the affected dogs, the daily dose could be tapered after four weeks to an every-other-day regimen and, in some cases, further reduced to twice-weekly administration. Dose tapering, achieved either by extending intervals between doses or by decreasing daily amounts, appeared to maintain similar clinical outcomes, with interval extension likely enhancing owner compliance [38,39,40,41].

Adverse effects during CsA therapy were found to be primarily mild gastrointestinal disturbances, most commonly intermittent vomiting, and the treatment was generally well tolerated [26,42]. The prevalence of bacterial infections in atopic dogs treated with cyclosporine was reported to be approximately 11%, whereas in dogs receiving cyclosporine for other immune-mediated disorders or following renal transplantation, the incidence was around 17% [43]. A comprehensive peer-reviewed analysis of 15 clinical trials indicates that long-term cyclosporine use in canine atopic dermatitis has a favorable risk–benefit profile. Most adverse effects were mild and manageable, primarily gastrointestinal, with vomiting reported in approximately 31% of cases and soft stools or diarrhea in about 20%. Less common events included decreased appetite, miscellaneous reactions, nodules or cysts, urinary tract infections, gingival hyperplasia, lethargy, reproductive disturbances, and papillomatosis, each occurring in roughly 1% of dogs. Rare events (<1%) comprised lymphadenopathy, neurological signs, urinary abnormalities, and hypersensitivity reactions. Only a small proportion of dogs required discontinuation of therapy due to adverse drug reactions [44].

The potential effect of administering cyclosporine on an empty stomach versus with food was evaluated in a two-way crossover study. Feeding at the time of drug administration achieved IL-2 expression levels consistent with adequate immunosuppression and appropriate blood drug concentrations [45]. Additionally, administration of a modified cyclosporine formulation with food did not appear to alter IL-2 gene expression or the expected peak plasma concentration. Consistent with prior reports, the most commonly observed adverse events in the study cohort were vomiting, diarrhea, and anorexia [45].

Evidence from multiple randomized controlled and open-label trials summarized in a review study indicates that cyclosporine provides robust long-term control of non-seasonal canine atopic dermatitis, markedly reducing pruritus and cutaneous lesion severity. Implementation of dose tapering, either via reduced daily doses or extended administration intervals, maintained therapeutic efficacy, with the majority of patients achieving good-to-excellent overall clinical outcomes. Collectively, these findings support cyclosporine as a recommended long-term management strategy for persistent canine atopic dermatitis, demonstrating sustained efficacy [46].

#### 3.2.2. Topical Use

In a double-blinded, randomized clinical trial, a lipophilic cyclosporine emulsion formulated for experimental investigation effectively controlled pruritus and reduced the severity of moderate-to-severe atopic dermatitis lesions in dogs. Clinical improvements were noted after 21 days of treatment and, by day 45, complete resolution of clinical signs was observed in 3 of 17 dogs receiving topical CsA. Importantly, no treatment-related adverse events were reported in any of the dogs. The rapid onset of effect of topical CsA, relative to oral administration, coupled with the absence of side effects, promoted high owner compliance in this study. Overall, topical CsA appeared to provide a safe and promising alternative for managing cAD in animals presenting with a limited number of localized lesions [47].

### 3.3. Mycophenolate Mofetil

Mycophenolate mofetil, an immunosuppressive agent with selective activity against lymphocyte proliferation, is utilized in both human and companion animal medicine for the management of immune-mediated conditions. In comparison with azathioprine, mycophenolate mofetil appears to be associated with a more favorable safety profile, particularly with regard to reduced risks of bone marrow suppression and hepatotoxicity [48,49].

In a recent study, the modified-release formulation of mycophenolate was investigated as a potential therapeutic alternative for dogs with moderate-to-severe atopic dermatitis. The findings suggested encouraging clinical potential. The therapeutic regimen of the formulation (30 mg/kg orally once daily) was initially determined from pharmacokinetic and pharmacodynamic studies in healthy dogs, yet further research is required to optimize dosing for canine atopic dermatitis and to define the most effective glucocorticoid co-administration strategy. Glucocorticoids were administered initially to achieve rapid symptom control, acknowledging that mycophenolate’s full effect may take several weeks. Early pharmacodynamic evidence indicates that the mycophenolate modified formula could induce a faster clinical response than immediate-release mycophenolate mofetil, although this remains to be confirmed. Response dynamics may also differ across organ systems. Observed rapid and sustained improvements in CADESI-04 scores suggest that a shorter glucocorticoid induction phase may be adequate, in line with contemporary recommendations favoring brief induction protocols when combining systemic immunomodulatory therapies [50].

### 3.4. Janus Kinase Inhibitors

#### 3.4.1. Oclacitinib

Oclacitinib acts through a mechanism that differs fundamentally from those of glucocorticoids and cyclosporine. When administered orally, it selectively targets Janus kinase 1 (JAK1), thereby inhibiting signaling pathways mediated by JAK1-dependent cytokines, including interleukin-31 (IL-31), which plays a central role in the transmission of pruritic stimuli to the brain [51,52].

Due to its rapid onset of action, often observed within hours, oclacitinib represents an effective option for the management of acute pruritus. Oclacitinib maleate exhibits rapid absorption in dogs and high oral bioavailability (79–89%), which is not influenced by food intake. Peak plasma concentrations are reached within one hour, and the elimination half-life ranges from 4.0 to 5.9 h, supporting both twice-daily and once-daily dosing regimens. Twice-daily administration for up to two weeks provides sustained inhibition of JAK1-dependent cytokines and results in significant improvement of pruritus and dermatitis, after which treatment may be transitioned to once-daily dosing for long-term management [24,52]. Extended twice-daily treatment with oclacitinib was associated with good tolerability and achieved clinical efficacy in most dogs. However, ongoing clinical follow-up and periodic blood testing were considered necessary when this regimen was employed [53].

Once-daily dosing achieves plasma concentrations above the IC_50_ for JAK1-dependent cytokines, effectively suppressing key pro-inflammatory mediators (e.g., IL-2, IL-4, IL-6, IL-13, and IL-31) while sparing non-JAK1-dependent cytokines involved in physiological processes such as hematopoiesis. This selective immunomodulatory effect reflects both the specificity of oclacitinib for JAK1 signaling pathways and the dosing regimen employed [52,54].

In a large randomized clinical trial, 436 dogs with allergic dermatitis and moderate-to-severe pruritus were allocated to receive either oclacitinib or placebo over a 28-day period. Treatment with oclacitinib at doses of 0.4–0.6 mg/kg administered twice daily resulted in a rapid therapeutic response, with clinical improvement observed within 24 h. Owner-evaluated pruritus visual analog scale (PVAS) [21,55] scores were significantly lower in the oclacitinib group than in the placebo group at all assessment time points (*p* < 0.0001). By day 7, a ≥50% reduction from baseline in pruritus, dermatitis, and PVAS scores was achieved in 70.5% of dogs receiving oclacitinib, compared with 23.2% of dogs in the placebo group. Overall, the findings demonstrated that oclacitinib provided a rapid and effective therapeutic benefit [56].

The drug is generally well tolerated and associated with a limited number of adverse effects [57]; however, it is not recommended for use in dogs under one year of age. Compared with glucocorticoids and cyclosporine, oclacitinib was associated with a lower incidence of adverse effects. In early clinical studies, the most frequently reported side effects included vomiting and diarrhea. Weight gain was observed more commonly than weight loss, while anorexia was reported in only 0.9% of treated dogs. Additional adverse events included demodicosis, pyoderma, and otitis in dogs receiving oclacitinib. Cutaneous lesions such as histiocytoma, papilloma, and other skin tumors were also reported in dogs treated with oclacitinib; however, their incidence did not appear to be increased when compared with dogs not receiving the drug (16.5% versus 12.8%) [24,52].

Current evidence suggests that the concomitant use of topical glucocorticoids with oclacitinib shortens the time to clinical remission and reduces the likelihood of rebound exacerbations [58]. The initial administration of oral prednisolone for four days significantly reduced the likelihood of pruritus rebound one week after oclacitinib dose reduction. This brief concurrent glucocorticoid treatment resulted in greater improvement of skin lesions and enhanced perceived treatment efficacy, while causing minimal adverse effects [59].

Although oclacitinib and lokivetmab are widely used in the management of canine atopic dermatitis, direct comparative data on their clinical efficacy remain limited. In one study, no significant differences were observed between treatment groups with respect to PVAS scores, and the magnitude of PVAS score reduction did not differ significantly between the two therapies. These findings indicate that both agents achieve comparable reductions in pruritus severity. While absolute CADESI-04 [21] scores were consistently higher in the lokivetmab-treated group across all evaluation time points, the degree of improvement in CADESI-04 scores over time was not significantly different between the two groups. Collectively, these results suggest that lokivetmab and oclacitinib produce similar clinical improvements in lesion severity, as measured by CADESI-04 [60]. It was also found that in dogs with insufficient response to monotherapy with oclacitinib or lokivetmab, oclacitinib–lokivetmab combination therapy may provide superior control of pruritus [61].

#### 3.4.2. Atinvicitinib

Atinvicitinib, approved in the European Union in 2025, is a second-generation, highly selective oral JAK1 inhibitor. It suppresses signaling from key pruritogenic and pro-inflammatory cytokines, including IL-31, IL-4, and IL-13, while showing at least tenfold lower activity against JAK2, JAK3, and TYK2. This selective JAK1 blockade may reduce interference with hematopoiesis and JAK2/JAK3-dependent immune functions compared with less selective agents such as oclacitinib or ilunocitinib, while maintaining rapid antipruritic effects. Clinical studies indicate that serological responses to core vaccines are preserved even at threefold the recommended dose, supporting the safe concurrent use of atinvicitinib with immunization [62].

A randomized, blinded, placebo-controlled trial evaluated atinvicitinib in 289 client-owned dogs with allergic dermatitis and moderate-to-severe pruritus across 26 US veterinary practices. Dogs received once-daily atinvicitinib (1.0 mg/kg; range: 0.8–1.2) or placebo for up to 28 days. Safety was assessed through adverse events and clinical pathology, and efficacy through daily owner-reported PVAS scores. Atinvicitinib was well tolerated, with only mild, transient adverse effects such as vomiting, diarrhea, reduced appetite, and lethargy. No papilloma or skin masses were reported, and hematology and clinical chemistry remained within reference ranges. Significantly more atinvicitinib-treated dogs achieved a ≥50% reduction in PVAS on at least 5 of 7 days compared with placebo, and by day 7, 81.8% of treated dogs had a ≥2 cm PVAS reduction versus 46.5% in the placebo group. These findings indicate that once-daily atinvicitinib is safe and effective for controlling pruritus in dogs with allergic dermatitis [63].

#### 3.4.3. Ilunocitinib

Ilunocitinib, a new Janus kinase inhibitor, is designed for the treatment of pruritus and skin lesions associated with allergic skin diseases in dogs. A six-month laboratory study using ilunocitinib tablets assessed the safety of once-daily dosing in healthy dogs. The study followed a randomized, blinded, parallel-group design, testing one to five times the maximum recommended dose (0.8 mg/kg) against sham-dosed controls [64].

Once-daily administration of ilunocitinib was well tolerated and led to rapid reductions in pruritus, with steady improvement over time. In a study comprising 306 dogs, 51.8% of treated animals achieved clinical remission of pruritus by day 28, regardless of the underlying allergic etiology [65].

Daily administration of ilunocitinib for six months at the recommended dose of 0.8 mg/kg, as well as at higher multiples up to 4.0 mg/kg, was well tolerated in Beagle dogs. Clinically relevant changes were absent at the therapeutic dose, while only minor alterations in hematological parameters, total protein, and fibrinogen were observed at elevated doses. These findings align with the expected pharmacology of JAK inhibitors and support safe long-term use [64].

Oral administration resulted in a Tmax of approximately 1–4 h, consistent with the rapid reduction in mean PVAS scores observed after the initial dose. The effect of food on ilunocitinib absorption was evaluated, as feeding can influence pharmacokinetics and owner compliance. Laboratory studies showed higher drug exposure under fed conditions, but field trials demonstrated consistent clinical efficacy whether tablets were given with or without food. These results indicate that ilunocitinib can be administered flexibly, without regard to meals, as exposure in fasted dogs is sufficient for therapeutic effect [66].

In a study comparing the efficacy and safety of ilunocitinib and oclacitinib for managing pruritus and skin lesions in dogs with atopic dermatitis, ilunocitinib rapidly and safely controlled clinical signs. It provided a significantly greater reduction in pruritus and lesion severity than oclacitinib, with a higher proportion of dogs achieving clinical remission of pruritus [67].

### 3.5. Lokivetmab

Recent investigations have increasingly emphasized the identification of novel therapeutic targets aimed at reducing reliance on broad-spectrum immunomodulatory agents, including glucocorticoids and cyclosporine, in favor of more selective treatment strategies, such as biologic therapies directed against key cytokines [39].

IL-31, a cytokine of the glycoprotein 130/IL-6 family, was first identified in 2004 and is produced primarily by Th2 cells after allergen presentation by Langerhans cells. It is also secreted by activated macrophages, basophils, eosinophils, and keratinocytes during allergen exposure or Type 2 inflammatory responses. Elevated IL-31 levels are associated with pruritic allergic skin conditions and induce scratching behavior. Lokivetmab, a caninized monoclonal antibody that neutralizes IL-31, is administered subcutaneously every four to eight weeks and is approved globally for the treatment of pruritus and other clinical signs of atopic and allergic dermatitis in dogs. Clinical studies demonstrate a significant reduction in pruritus as early as day 1 and improvement in skin condition by day 7 [68,69].

In a study regarding the effects of lokivetmab, a significant reduction in PVAS scores and improvements in quality of life for both dogs and owners were observed, with PVAS positively correlating with the quality of life of both groups. On the other hand, hair cortisol levels were significantly lower on day 56 compared with day 28, suggesting its potential as a biomarker of stress in dogs with chronic atopic dermatitis. These findings highlight the negative impact of cAD on the quality of life of both dogs and their owners, while demonstrating the beneficial effects of lokivetmab therapy [70].

A study conducted to further evaluate the onset and duration of the antipruritic effect of a single subcutaneous injection of lokivetmab reported that a 2 mg/kg dose produces a significant reduction in pruritus, beginning 3 h post-administration, which is maintained for up to 42 days [68]. In addition, the monthly dosing regimen eliminates the need for daily home treatment, enhancing convenience and leading to high owner satisfaction. However, the relatively high cost and the necessity for ongoing long-term therapy may limit its accessibility despite demonstrated efficacy and positive owner experiences [71].

The results of a study indicate that IL-31 neutralization with lokivetmab provides effective control of pruritus across a broad population of dogs, not limited to those with a confirmed diagnosis of chronic atopic dermatitis [72,73]. Lokivetmab partially controls cutaneous inflammation, as it targets only IL-31-mediated pathways, leaving other inflammatory mechanisms largely unaffected; therefore, combination with other therapies is sometimes required to achieve broader control of the disease [72].

In a study regarding the safety of lokivetmab, reported adverse effects included vomiting (15.5%), diarrhea (13.4%), lethargy (9.9%), anorexia (4.9%), bacterial skin infection (7%), pruritus (5.6%), and otitis externa (5.6%). Three dogs experienced transient pruritus, *Malassezia* dermatitis, and generalized lymphadenopathy, respectively, during treatment. Notably, generalized lymphadenopathy has not previously been described as a side effect of lokivetmab. The mechanism is not fully understood but may involve IgE-mediated responses to the 5% murine polypeptide component [60].

In a prospective study, the potential of topical products containing plant extracts to enhance the clinical efficacy of lokivetmab was evaluated, suggesting that combining lokivetmab with therapies aimed at repairing the skin barrier may improve treatment outcomes in dogs with atopic dermatitis [74].

### 3.6. Allergen-Specific Immunotherapy (ASIT)

Allergen-specific immunotherapy (ASIT) consists of the controlled administration of increasing concentrations of allergen extracts to allergic patients in order to reduce clinical reactivity upon natural exposure. The dosing regimen is gradually escalated until a maintenance dose or an individualized maximum tolerated dose is reached. This treatment modality has been variously described as desensitization, hyposensitization, or immunotherapy in previous studies [75,76].

As allergen-specific immunotherapy is considered to modify the underlying immunological mechanisms of allergic disease, it represents the only therapeutic approach with the potential to alter the natural course of allergy and prevent the progression to additional hypersensitivities. ASIT offers the prospect of long-term remission and may allow for treatment regimens with relatively infrequent administration, particularly when compared with oral therapies that require daily or multiple daily dosing. In contrast, achieving and maintaining sustained clinical control of atopic dermatitis using alternative strategies, such as allergen avoidance or symptomatic treatment alone, can be challenging. Moreover, life-threatening adverse reactions associated with ASIT are rarely reported, and no long-term adverse effects have been documented in dogs, suggesting that few contraindications exist for its use [76].

ASIT differs from symptomatic treatments in that it targets the underlying immunological mechanisms of atopic dermatitis, rather than merely controlling flares or pruritus. By modulating the immune response, ASIT has the potential to alter the natural course of the disease and induce long-term remission. Symptomatic therapies, in contrast, provide rapid relief of clinical signs but do not modify disease progression, highlighting the complementary roles of these approaches in comprehensive management.

Treatment duration was strongly associated with the clinical efficacy of allergen-specific immunotherapy. Animals receiving ASIT for less than 12 months exhibited lower response rates (22%) compared with those treated for at least 12 months (65%). Additionally, in dogs treated for 12 months or longer, the need for concomitant medications was reduced to a greater extent (87%) than in those treated for shorter periods (39%) [77].

Rush immunotherapy, administered through a condensed induction period, often completed in a single day at a veterinary hospital or specialist practice, provides a safe and effective approach for managing canine atopic dermatitis. This protocol allows for faster amelioration of clinical signs while reducing the risk of dosing errors and confusion. By minimizing the number of required clinic visits, this version of immunotherapy also promotes owner compliance, a key factor in the success of long-term immunotherapy. Observations from human immunotherapy support the benefit of shortening the induction phase and decreasing injection frequency to improve adherence. Nevertheless, further randomized (and, ideally, blinded) studies with larger patient populations are needed to compare the efficacy of rush with conventional immunotherapy [78,79].

Subcutaneous allergen-specific immunotherapy has been shown to improve clinical signs by at least 50% in approximately 59.9% of dogs with atopic dermatitis. Treatment outcomes appear to be more favorable when patients are monitored regularly and when the use of long-term systemic corticosteroids is avoided, particularly during the initial nine months of ASIT [80]. In a large clinical study, sublingual immunotherapy (SLIT) demonstrated a success rate of approximately 55%, comparable to that reported for subcutaneous immunotherapy (SCIT). The oral mucosa contains fewer pro-inflammatory cells, such as mast cells and eosinophils, which reduces the likelihood of allergic inflammation and contributes to the favorable safety profile of SLIT [75].

Both SCIT and intralymphatic immunotherapy (ILIT) have been shown to improve clinical signs of canine atopic dermatitis, with ILIT demonstrating a notably higher rate of return to normal [81]. The efficacy of ILIT, however, appears to depend on the quality of injections, as low-quality or improperly delivered injections may contribute to treatment failure. These findings highlight the importance of performing ILIT under ultrasound guidance [82] by trained personnel to ensure accurate intralymphatic delivery and optimize therapeutic outcomes, while still benefiting from the advantage of a reduced number of injections compared to conventional SCIT [83].

Lack of owner compliance was identified as the primary factor contributing to reduced ASIT effectiveness. These findings underscore the importance of providing veterinarians and owners with adequate education regarding the mechanisms of action and expected outcomes of ASIT prior to treatment initiation in order to improve adherence and therapeutic success [77].

Recurrence of clinical signs following discontinuation of allergen-specific immunotherapy has been reported in humans, supporting the recommendation of continued monitoring for relapse. In a case report, the use of sublingual immunotherapy was associated with complete remission of canine atopic dermatitis without observed adverse effects. These findings indicate that SLIT may represent an effective and safe option for the long-term management of cAD [75].

The findings of another study further support the clinical and immunological benefits of allergen-specific immunotherapy in dogs affected by chronic atopic dermatitis. A favorable clinical response to ASIT was observed in the majority of treated animals, with six out of seven dogs demonstrating improvement following therapy. In addition to clinical efficacy, ASIT was associated with marked immunomodulatory effects, reflected by changes in both lymphocyte subpopulations and circulating cytokine profiles in the peripheral blood of treated dogs. Specifically, reductions in pro-inflammatory cytokines such as IL-13 and TNF-α were observed, alongside alterations in T cell populations, including a decrease in cytotoxic T lymphocytes and an increase in activated T cells. These immunological shifts are consistent with mechanisms that may underlie the therapeutic efficacy of ASIT in modulating allergic inflammation. Furthermore, the duration of immunotherapy appeared to influence the number of regulatory T cells, suggesting a time-dependent immunological adaptation. Although a transient decrease in Treg cell counts was noted after three months of therapy, levels subsequently increased after six months to values comparable to or exceeding baseline levels; however, the biological significance of this fluctuation remains unclear. It should be emphasized that despite the consistent trends observed at the group level, individual responses to ASIT varied considerably among dogs. This interindividual variability highlights the complexity of immune regulation in atopic dermatitis and underscores the importance of individualized monitoring when evaluating therapeutic outcomes of allergen-specific immunotherapy [84].

### 3.7. Complementary Therapeutic Approaches

In cAD, a likely defective skin barrier allows allergens to penetrate and initiate abnormal immunological reactions [69,85,86]. Skin barrier dysfunction is recognized as a central element in the pathogenesis of canine atopic dermatitis, although it remains debated whether it represents a primary defect or arises secondary to inflammation. This dysfunction is characterized by multiple alterations, including changes in stratum corneum proteins, modifications of the skin lipid profile, and imbalances in the cutaneous microbiome similar to those from human atopic dermatitis (Figure 2) [87]. However, the current body of evidence remains limited, as available studies are relatively few and often involve small cohorts of animals, indicating that this therapeutic approach is still in an exploratory stage.

Franco et al. [88] showed that lipid abnormalities associated with canine atopic dermatitis extend beyond the stratum corneum and inflamed epidermis, as similar alterations were also detected in the blood and in non-lesional skin, albeit with less pronounced effects. These observations support the concept of cAD as a systemic disorder characterized by widespread dysregulation of lipid metabolism [2].

Evaluation of topical emollients in dogs remains limited, but a newly developed emollient for atopic dogs showed significant clinical benefits in a clinical trial involving 21 dogs with non-seasonal AD. Treatment led to reductions in pruritus and lesion severity, with mean pVAS10 scores decreasing from 4.25 to 3.38 and 81% of dogs showing improvement. The proportion of dogs with “normal” or “mild” pruritus increased from 33% at baseline to 71% after 30 days, supporting the effectiveness of topical emollients in managing key features of canine atopic dermatitis [87].

In a study concerning acute barrier disruption in healthy dogs, weekly topical applications of a formulation containing plant-derived essential fatty acids and essential oils effectively mitigated increases in transepidermal water loss and preserved skin hydration, suggesting its potential as an adjunctive therapy for skin barrier defects. Further studies are required to confirm these effects as the relatively small cohort in this study may have limited the statistical power and generalizability of the findings [85].

An ideal topical formulation for canine atopic dermatitis should be emollient, support the lipid barrier, help balance the skin microbiome, and easy to apply, well-tolerated, and safe for both the dog and the owner, with some residual activity. Ophytrium, a novel compound derived from *Ophiopogon japonicus* (Japanese mondo grass), has been shown to inhibit inflammatory pathways, preserve epidermal structure and barrier function, and reduce staphylococcal adherence and biofilm formation in both human and canine epidermal models. In an open pilot study, it has been incorporated into shampoos and foams for dogs, with treatment protocols involving 2–3 cycles of one shampoo followed by foam applications every 2–3 days, demonstrating improvements in cAD and superficial staphylococcal pyoderma (Ophytrium/3% chlorhexidine). The use of effective topical therapies, whether alone or in combination with other treatments, can enhance both the safety and efficacy of cAD management, while also reducing reliance on systemic antimicrobials, an important consideration for antimicrobial stewardship. The fact that the current evidence is derived from open-label pilot studies underscores the need for rigorous, double-blind, placebo-controlled trials to definitively establish efficacy [89].

Marsella et al. [90], in a double-blinded, controlled clinical trial, investigated the potential benefits of glycosaminoglycans (GAGs)—particularly hyaluronic acid—due to their effects on skin hydration and barrier integrity. The research assessed a topical formulation combining sphingolipid and GAG extracts in dogs with atopic dermatitis. This formulation was distinct from previously tested products because the sphingolipid component was enriched in sphingomyelin, a ceramide precursor which has been shown in vitro to enhance endogenous ceramide synthesis and promote the lamellar structures essential for maintaining epidermal barrier function. It was hypothesized that the combination of sphingomyelin-rich sphingolipids with GAGs could improve both the clinical severity of cAD and skin barrier function. The results of the study demonstrated that the topical formulation mitigated clinical exacerbations induced by house dust mite exposure, leading to reductions in pruritus and lesion severity. These findings indicate that the formulation may be effective as an adjunctive therapy to conventional treatments and, in selected cases, as a standalone intervention for mild to moderate AD. By supporting barrier function and potentially modulating inflammatory responses, this sphingolipid and GAG-enriched topical formulation represents a promising approach for the management of canine atopic dermatitis.

Cannabidiol (CBD) and cannabidiolic acid (CBDA) have been reported to possess antinociceptive, immunomodulatory, and anti-inflammatory properties, making them of interest as potential adjunct therapies for canine atopic dermatitis. In a randomized, double-blinded and placebo-controlled trial investigating their clinical effects, administration of CBD/CBDA did not produce significant changes in skin lesion severity but was associated with a reduction in pruritus in some treated dogs. These findings highlight the ongoing exploration of novel therapeutic strategies aimed at improving the management of atopic patients, including the development of topical or adjunctive treatments that can complement existing systemic therapies and provide additional relief from pruritus while minimizing adverse effects [91].

Dysbiosis is commonly observed in dogs with atopic dermatitis, and antimicrobial therapy may be required during disease flare-ups. Topical interventions can help prevent the development of bacterial resistance and antiseptic wipes offer a simple method for local application. A multicentric, prospective, open-label study carried out by three specialists from the European College of Veterinary Dermatology on 3% chlorhexidine-impregnated wipes demonstrated that the wipes effectively managed local areas of dysbiosis in dogs with cAD, reducing the need for systemic medications and helping to limit the risk of bacterial resistance [92]. Emollient bathing products may be effective in managing *Malassezia* overgrowth in dogs with atopic dermatitis, as has been reported in a randomized, single-blinded trial. However, even when using emollient formulations, frequent bathing with shampoo can potentially impact skin barrier function [93].

Noli et al. [94], in a prospective, open-label, multi-center, 14-day clinical trial evaluating the topical management of canine atopic dermatitis by daily application of a mousse containing adelmidrol, tapioca starch, and an antimicrobial complex, combined with gentle shampooing, demonstrated that the mousse was successful in reducing seborrhea and associated clinical signs. Improvements in pruritus, skin lesions, and microbial overgrowth were observed, supporting the potential of this approach as an effective adjunctive therapy.

The potential benefits of topical heat-treated bacteria for managing canine atopic dermatitis were explored in a preliminary study involving 10 client-owned dogs, which reported a significant reduction in both clinical scores and pruritus, which persisted for up to two weeks after treatment cessation, suggesting a residual therapeutic effect. Owner feedback indicated that the product was generally considered easy to use, ranging from easy to very easy, and effective in controlling the clinical manifestations of cAD. Notably, improvements were observed not only in the areas directly treated but also in regions such as the feet and head, which were not the primary targets of application. Despite these clinical improvements, analyses of the cutaneous microbiota revealed no detectable changes after 28 days of daily application, indicating that the observed benefits may be mediated through mechanisms other than direct microbiome alteration. These findings suggest that topical administration of heat-treated bacteria may represent a safe, user-friendly adjunctive strategy to alleviate pruritus and reduce clinical signs in dogs with atopic dermatitis, even in areas beyond those directly treated [95].

Taken together, these findings support the growing relevance of barrier and microbiome-targeted interventions as adjunctive components in the management of canine atopic dermatitis, with demonstrated potential to improve clinical signs, enhance skin integrity, and reduce reliance on systemic therapies. However, the current evidence base remains limited, as many of the available studies are characterized by small cohort sizes, heterogeneous study designs and, in several cases, open-label or preliminary methodologies. Consequently, while these approaches appear promising, their efficacy and long-term benefits cannot yet be considered fully established.

Further well-designed, large-scale, randomized controlled trials are therefore required to validate these preliminary observations, clarify the associated mechanisms of action, and establish standardized, evidence-based protocols. Continued research in this area will be essential to determine the true clinical value of topical and barrier-focused therapies and to define their optimal integration into comprehensive management strategies for canine atopic dermatitis.

## 4. Conclusions

The management of canine atopic dermatitis increasingly reflects the necessity of a multidimensional approach, in which systemic immunomodulation, cytokine-targeted therapies, and barrier-focused interventions collectively influence clinical outcomes. The transition from broad-spectrum immunosuppressants to more targeted therapies, including Janus kinase inhibitors and IL-31 monoclonal antibodies, represents an effort to refine therapeutic precision; however, translating these targeted effects into consistent and durable disease control remains a clinical challenge. In practice, systemic glucocorticoids retain an important role in the short-term management of acute, highly pruritic, and inflammatory flares, whereas cyclosporine is more appropriately positioned as a long-term immunomodulatory option due to its delayed onset of action. Janus kinase inhibitors provide rapid antipruritic effects with additional anti-inflammatory activity and may be used in both short- and long-term settings with appropriate monitoring. Lokivetmab is particularly effective in patients in which pruritus predominates, although its more selective mechanism often necessitates combination with other therapies or its use as a maintenance strategy following initial disease control.

Allergen-specific immunotherapy remains the only intervention with clear disease-modifying potential, particularly in non-seasonal atopic dermatitis, but its efficacy is influenced by treatment duration and patient compliance. Due to its delayed onset of action, concurrent symptomatic therapy is typically required to control pruritus and inflammation during the initial phases of treatment. The variability in therapeutic response further highlights the need for individualized treatment strategies and suggests a potential role for biomarkers in guiding therapy selection.

Adjunctive strategies aimed at supporting the skin barrier and modulating the cutaneous microbiome underscore the contribution of local processes to overall disease expression and may enhance the effects of systemic therapies. While such interventions can contribute to clinical improvement, they are best regarded as complementary rather than stand-alone treatments. In addition, the relative scarcity of robust, high-quality studies, which are often limited by small cohorts, emphasizes the need for further investigation to establish standardized, evidence-based protocols. Consequently, optimal management relies on the careful integration and sequencing of available therapies, tailored to the individual patient, and may require iterative therapeutic trials to achieve sustained efficacy while minimizing adverse effects.

## Data Availability

The original contributions presented in this study are included in the article. Further inquiries can be directed to the corresponding author.

## Abbreviations

The following abbreviations are used in this manuscript:

ASIT	Allergen-Specific Immunotherapy
cAD	Canine Atopic Dermatitis
CADESI	Canine Atopic Dermatitis Extent and Severity Index
CBD	Cannabidiol
CBDA	Cannabidiolic Acid
csA	Cyclosporin A
GAGs	Glycosaminoglycans
IFN	Interferon
IgE	Immunoglobulin E
IL	Interleukin
ILIT	Intralymphatic Immunotherapy
JAK	Janus Kinase
PVAS	Pruritus Visual Analogue Scale
SCIT	Subcutaneous Immunotherapy
SLIT	Sublingual Immunotherapy
TNF	Tumor Necrosis Factor
TYK	Tyrosine Kinase
